# Esophageal Cancer Metastases to Unexpected Sites: A Systematic Review

**DOI:** 10.1155/2017/1657310

**Published:** 2017-06-04

**Authors:** Osama Shaheen, Abdulaziz Ghibour, Bayan Alsaid

**Affiliations:** ^1^Department of General Surgery, Damascus University Hospitals, Faculty of Medicine, Damascus University, Damascus, Syria; ^2^Laboratory of Anatomy, Faculty of Medicine, Damascus University, Damascus, Syria

## Abstract

The most common pattern of esophageal cancer metastases (ECM) is to the lymph nodes, lung, liver, bones, adrenal glands, and brain. On the other hand, unexpected metastasis (UM) spread to uncommon sites has increasingly reported and consequently affected the pathway of diagnosis, staging, and management. Using the PubMed database, a systematic search of the following headings “Esophageal” and “Metastasis” or “Metastases” was performed, 10049 articles were identified, and the articles were included if they demonstrated unexpected ECM. 84% of cases were men with an average age of 60.7 years. EC was located in the lower third in 65%. Two-thirds of the UM originated from the lower esophagus, and the two major histological types were adenocarcinoma 40% and squamous cell carcinoma 60%. Metastases were disseminated toward five main anatomical sites: the head and neck (42%), thoracic (17%), abdomen and pelvis (25%), extremities (9%), and multiple skin and muscle metastases (7%). The EC metastases were found to be synchronous 42% and metachronous 58%, isolated in 53.5% and multiple in 46.5%. The overall survival rate was 10.2 months. Since distant metastases are responsible for most EC-related deaths, understanding of ECM dissemination patterns needs more extensive studies. These critical data are the cornerstone of optimal cancer approach and treatment.

## 1. Introduction

Esophageal cancer (EC) is one of the exceedingly aggressive cancer worldwide; hence, relatively, a scant number of articles was published trying to study EC distinctive features. The overall incidence of EC in the United States has been fairly stable for many years, with 5-year relative survival rate ranges between 40% for localized tumor (N0-M0) and 4% for advanced distal metastasis tumors (M1, [1-2]). Since distant metastasis represents the most common cause of cancer-related death, secrets of EC cell dissemination constitute the most valuable key in optimal approaching of EC features.

Up until now, several studies have investigated the complexity of EC lymphogenic-hematologic metastasis patterns [3–5]. Clearly, EC locoregional and expected distal metastasis features have been widely studied. However, a distinct number of clinical articles have reported on the frequency of the unexpected esophageal cancer metastases (ECM). Correspondingly, we reviewed and summarized the scientific literature on the unexpected ECM distribution, with the goal of achieving a closer insight about the tumor cell distribution principles, and help determining optimal cancer control strategies.

## 2. Methods

### 2.1. Study Population and Search Strategy

Between 1982 and February 2017 using the PubMed database (US National Library of Medicine, Bethesda, Maryland), a systematic medical literature search was conducted by the researchers to identify the articles describing uncommon ECM.

**Figure 1:**
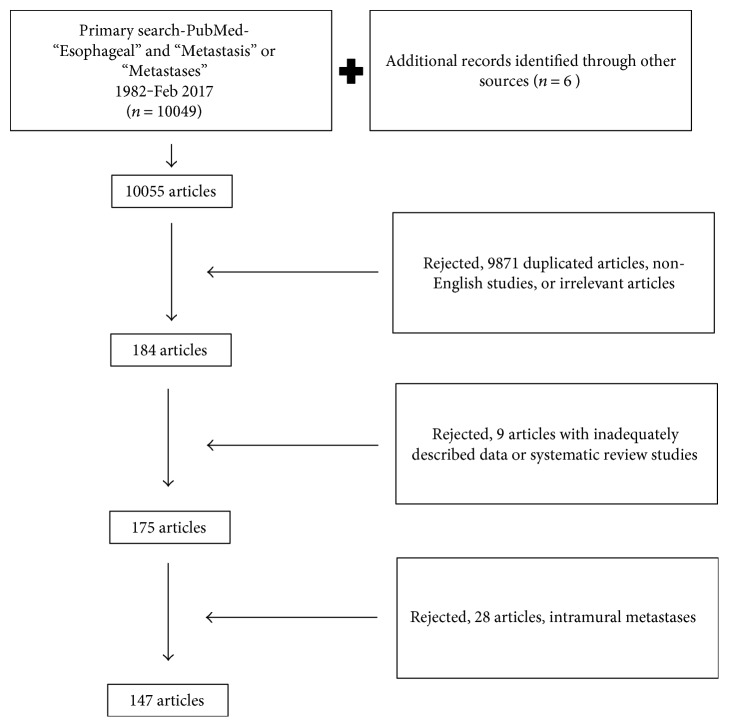
The selection process of the study.

First, using the following key words “Esophageal” and “Metastasis” or “Metastases”, 10049 articles were identified and additional 6 cases from other resources were identified and included. Second, the titles and abstracts of these articles were reviewed, the articles with expected metastases were dropped from the study, and the articles were included if they demonstrated case report or case series of unexpected ECM. Eventually, relevant articles were reviewed and sorted for final analyzation.  Figure 1() represents the flow diagram for searching and extracting data (between 1982 and February 2017).

### 2.2. Selection Criteria

The systematic review started from a very broad search process to include every possible article. The next step limited our search only to English language original articles, case reports, case series, or editorial letters that described the unusual locations of ECM. Each of these organs was considered common metastasis sites and was dropped from the study: the liver, bone, lungs, adrenal glands, and brain. Since it has specific spread rout, the articles who described intramural metastases were also added to the exclusion criteria.

### 2.3. Selection of Data

The authors selected studies based on the titles or abstracts. Studies that met the inclusion criteria were selected for review. If it was not clear from the abstract whether a study fulfilled the inclusion criteria, the full article was retrieved for further evaluation.

### 2.4. Data Extraction and Quality Assessment of Included Material

In total, 164 cases (out of 147 articles) [6–153] of unexpected ECM were selected to establish our database. The following data elements were extracted from each article: author name, publication year, article type, patient sex, age at diagnosis, histological type of cancer, tumor location in the esophagus, stage at diagnosis, management of primary tumor, site of metastases, metastasis features (solitary, multiple, synchronous, metachronous, and onset after initial tumor diagnosis), management of metastases, outcomes, and survival.

Extracted data were treated in Excel table and validated by the three authors.

## 3. Results

### 3.1. Patient Characteristics

Between 1982 and February 2017, a total number of 164 patients were included in the study. Clinicopathological characteristics of EC with unexpected metastases were summarized in Table 1. In general, 84% of the patients were male, 16% female, and the median age at diagnosis was 60.7-year-old with almost half of these cases were designated as stage IV upon the initial diagnosis. About two third of the unexpected metastases originated from the lower esophagus, and the two major histological types of the cancer were adenocarcinoma in 40% and squamous cell carcinoma in 60%.

### 3.2. Anatomical Distribution of Unexpected Esophageal Cancer Metastases

**Figure 2:**
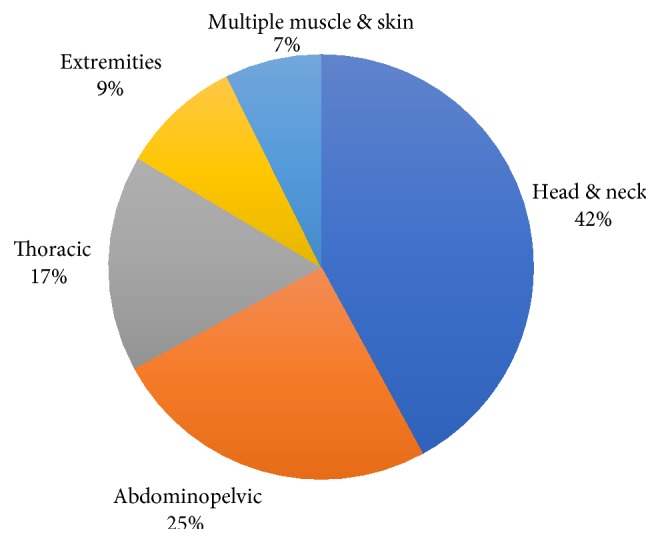
Unexpected ECM stratification according to anatomical site spread.

**Figure 3:**
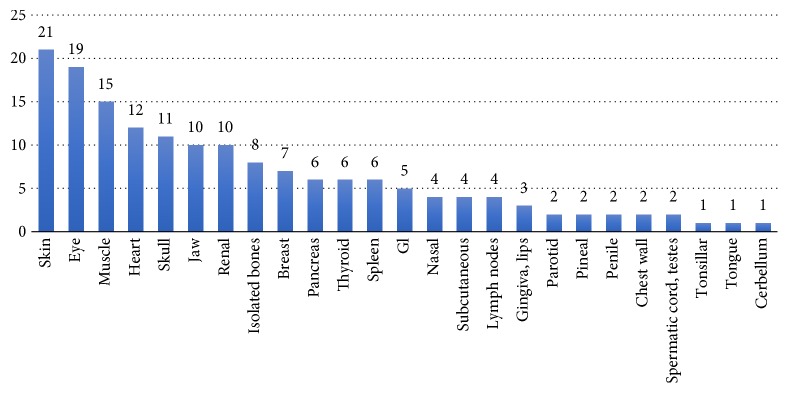
Unexpected ECM trend across organs.

Unexpected ECM tend to spread to different anatomical sites. For statistical analysis purposes, metastases were stratified according to five main anatomical sites (groups), the head and neck, abdominopelvic, thoracic, extremities, and multiple skin and muscle metastases (Figure 2). On the other hand, Figure 3 illustrates a different analytic scenario according to organ metastasis. For example, the skin, eye, muscle, and heart represented the most common (13%, 12%, 9%, and 7%, resp.) organ metastases while the tonsillar, tongue, and cerebellum were scarcely reported (only one case each).

### 3.3. Esophageal Cancer Features across 5 Major Metastasis Anatomical Groups

In this study, we have evaluated the frequency of many EC primary features among metastasis major location.

**Figure 4:**
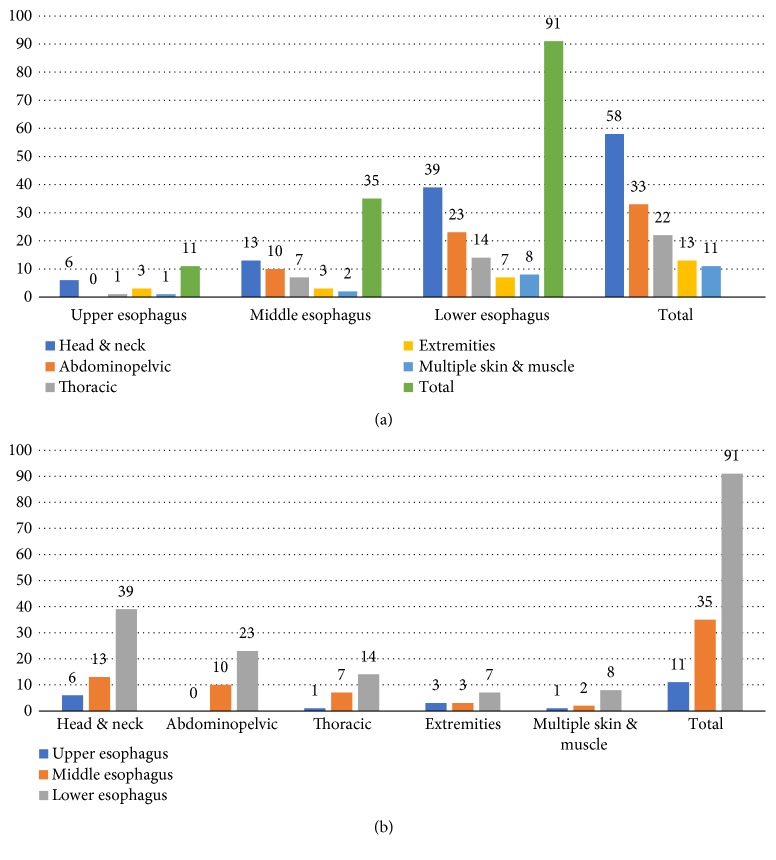
(a) Primary EC location influence on metastasis distribution. (b) Metastasis trend according to primary EC location.

First, regarding general metastasis anatomical groups, as illustrated in Figure 4, the head and neck metastases represented the most common unexpected metastases (42%), with lower esophagus considered as the most frequent initial tumor location (66%). On the other hand, upper EC constituted only 8% of initial tumor locations with the head, neck, and extremity regions represented 90% of its metastases.

Second, regarding organ metastasis stratification, in thoracic group, the heart and breast constitute the most common sites of UM, in abdominopelvic cavity group; UM were most common to the renal, pancreas, and spleen, in head and neck group; spread was most common to the eye, jaw, skull, and dura (Figure 3).

**Figure 5:**
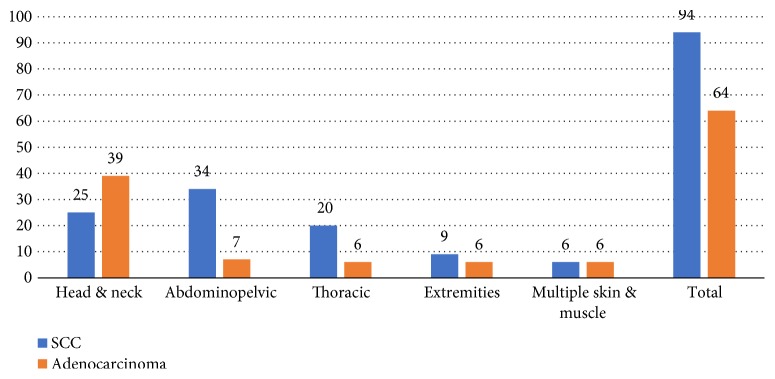
ECM anatomic location influence on pathological rate.

Third, considering tumor histological classification; as elucidated in Figure 5, SSC constituted 60% of cases in general. In the abdominopelvic and thoracic groups, SCC represented over 75% of cases. However, adenocarcinoma represented 60% of cases in the head and neck group and 50% in the multiple skin and muscles group.

**Figure 6:**
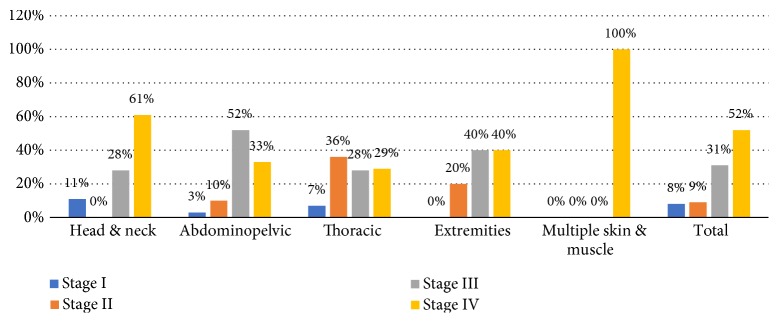
Tumor stage trend upon diagnosis among different ECM anatomical locations.

Fourth, the relation between metastasis anatomical groups and initial cancer stage was demonstrated in Figure 6, and it showed that in general, 83% of cases were initially diagnosed as stage III or IV. However, in the thoracic group, 42% of cases were stage I or II, and in multiple metastasis group, 100% of cases were stage IV.

### 3.4. Cancer-Metastasis Synchronizations

**Figure 7:**
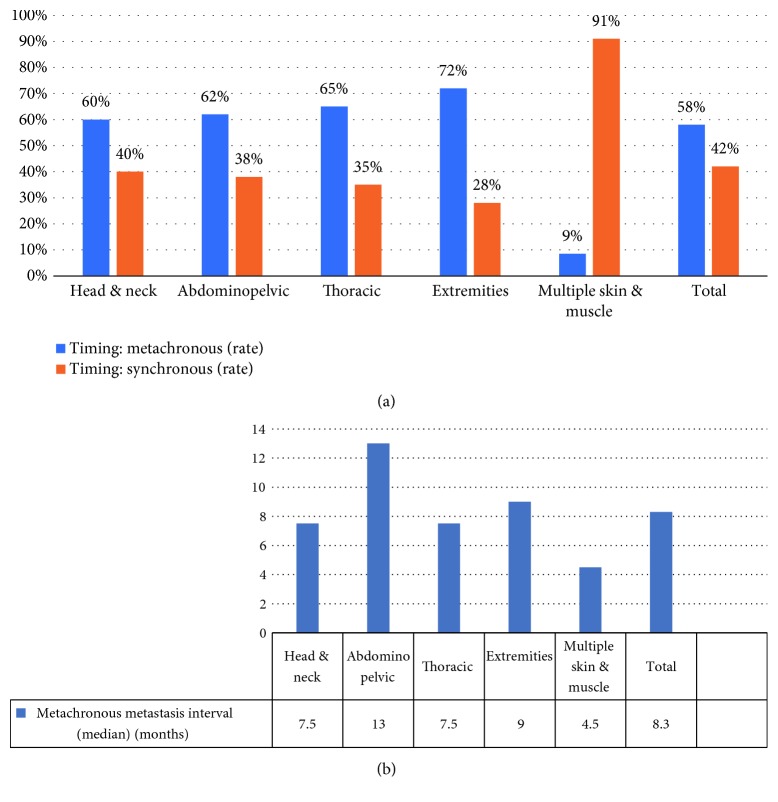
(a) Cancer-metastasis onset (metachronous and synchronous) across ECM anatomical groups. (b) EC and metastasis interval (median and months).

As an important variable, we investigated the probability of cancer-metastasis synchronization in different anatomical groups. As shown in Figure 7, in general, metastases were diagnosed at the same time with the initial tumor in 42% of cases, and interestingly, this rate approaches 91% in multiple metastasis group and sometimes were the first symptoms of the tumor. On the other hand, metastases were metachronous in 58% in cases (72% in the extremities group) and the median interval time between initial tumor and metastasis diagnosis was 8.3 months (4.5 months in the extremity group versus 13 months in the abdominopelvic group).

### 3.5. Metastasis Approaching and Lifetime Prognosis

**Figure 8:**
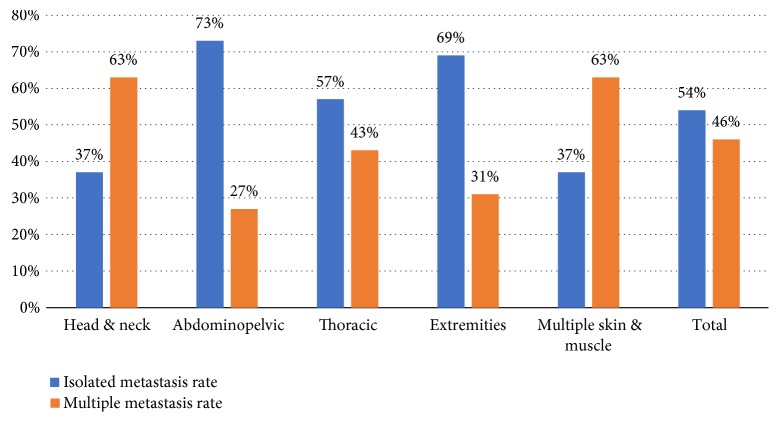
Metastasis features (isolated versus multiple).

Finally, we investigated the metastasis evaluation process and the influence of the metastasis location on lifetime prognosis. It was important to know (as shown in Figure 8) that 54% of the UM were isolated upon diagnosis and this trend reached 70% in the extremity and abdominopelvic groups; on the other hand, this rate was only 37% in the head and neck group.

**Figure 9:**
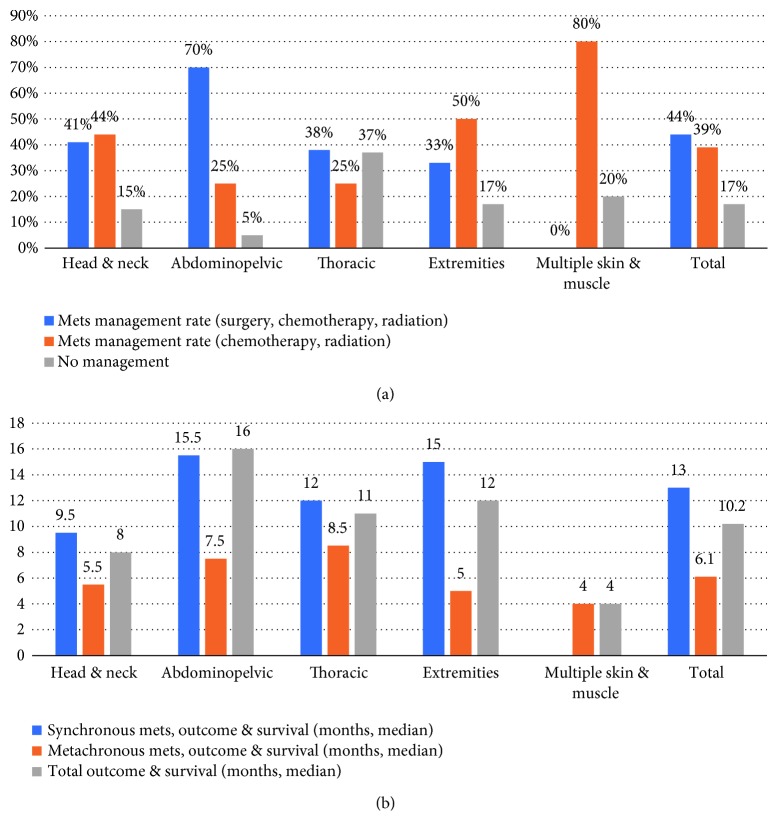
(a) Metastasis management. (b) Outcome and survival.

The analysis of the metastasis management plan based on location was demonstrated in Figure 9 and notably showed that in general, surgery was part of the management plan in 44% of cases and no management was applicable in 17% of cases. However, when considering anatomical groups, surgery was available in 70% of the abdominopelvic group and no management was applied in 37% of the thoracic group.

The influence of both metastasis anatomical location and cancer-metastasis synchronization on the total outcome was shown in Figure 9. Regarding synchronization, the survival rate was 13 months in synchronous group versus 6.1 months in the metachronous group. From a different standpoint, survival rate approached 16 months in the abdominopelvic group versus 4 months in the multiple skin and muscle metastasis group.

## 4. Discussion

Historically, cancer-distal metastases have been always considered the most potential barrier in achieving a significant advance in cancer management. In addition, being responsible for 90% of cancer-related deaths [154, 155], the question of how tumor cells metastasize across different anatomical sites has the key role in establishing optimal cancer-approaching plan.

Remarkably, the impact of esophageal cancer regional and usual distal metastases on survival and outcome has been widely studied in various, well-controlled studies. On the other hand, unexpected metastases have been only investigated through sporadic case reports and small case-series studies.

Here, in this study, we have determined esophageal cancer-unexpected metastasis features and have investigated their clinicopathological variations across different anatomical site distribution.

Our study demonstrated that EC has a special tendency for unexpected specific site expansion, and this behavior has been attributed to the unique anatomical esophageal features which considered a key player in elucidating EC distinctive and extremely aggressive nature.

As already known, the absence of serosal coating and the presence of periesophageal adventitia that connects it with the mediastinum structures including the recently discovered aorto-esophageal ligament [156] have an important impact on the lymph node metastasis frequency and tumor ingrowth into the surrounding and distal organs.

These unique features can also be partly attributed to its multiple arterial resources (shared vasculature), starting from the inferior thyroid artery to the splenic artery across different arterial supplies between them. Moreover, the esophagus extrinsic veins drain into the locally corresponding large vessels (the jugular veins or the azygos and hemizygous veins superiorly and to the left gastric and splenic veins inferiorly). Interestingly, having penetrated the muscular wall, the small vessels form abundant submucosal plexus which could explain the existence of distal unexpected metastases in some patient with low-stage cancer (1 or 2).

Furthermore, the complex anatomical pathway of the esophagus lymphatic network including the lymphatic nodal skip (retrograde and bidirectional) spread [156–158] can also explain the possibility of the random distribution of the metastases in esophagus cancer.

In this context, based on hematologic and lymphatic anatomy, EC distant metastases can leave the esophagus via three possibilities: lymphatic, venal, or arterial routs. The venal routes will pass in the vena cava then the pulmonary system explaining the standard pulmonary metastases, or in the portal system explaining the hepatic metastases [159]. However, the isolated distal metastases in terminal organs or anatomical structures such as the skin, penis, lips, or retina can be hardly explained based on lymphatic or venal routs alone. Even though tumors almost always metastasize through the veins rather than through the arteries, the expected route of those unexpected metastases may be explained by the arterial pathway. In support of the former possibility, arterial blood has been proved to be a better source of circulating tumor cells than venous blood [160].

The lymphatic route could explain the invasion to the neighboring anatomical structure such as the neck, mediastinum, or gastric localization. The phenomena of retrograde lymph drainage due to intramural lymph vessel obstruction [161] could explain some distal intramural metastases; however, the distal isolated metastases are still poorly supported via this route.

In our opinion, the arterial route could be the way in which this tumor embolism liberates and pass toward the main artery to follow an anatomical direction and finally reach the distal terminal organs. Our observations could reinforce this proposition as some metastases were isolated, metachronous with no recurrence of the primary tumor. This anatomical hypothesis can be added to different beyond anatomical hypotheses where either certain molecular features acquired during tumor evolution serve as one master key for distinct locks or the microenvironment of two host organs resemble each other thereby equally facilitating metastasis in two anatomically distinct locations [162].

Our observations came to support many important previous studies. For instance, Bruzzi et al. have clearly illustrated the importance of integrated CT-PET in detecting distant esophageal cancer metastases that can be unusual in appearance and in unexpected locations [163]. Moreover, Nguyen et al.'s results have showed (using true whole-body F-18 FDG PET/CT) that esophageal cancer had a prevalence of 7.7% of unexpected soft tissue metastasis representing a higher prevalence when compared to lymphoma or lung carcinoma; furthermore, this study has also hypothesized that limited scanning may underestimate the true extent of soft tissue metastasis since a significant percentage of these metastases (46%) occurred outside the typical limited whole-body field of view [164]. Finally, our findings of higher possibility of isolated UM but still poor overall survival rate can also clarify what Türkyilmaz et al.'s study showed that current staging techniques alone fail to detect disseminated tumor cells in EC since survival rate were similar in patients with hematogenous metastases in different organs, whether or not they received chemotherapy [165].

Although this review is the first with this number of data, it has many limitations concerning the homogeneity of data. But it highlights a critical point in the EC. In summary, our current work of unexpected ECM across major anatomical sites strongly supports other basic and clinical studies in understanding the possible pathways of metastatic progression which have a great impact on effective cancer control strategies. Multicentric studies, experimental studies in vivo, and anatomical studies are demanded to clarify this unexpected ECM.

## 5. Conclusion

Since esophageal cancer-unexpected metastases can occur following any cancer stage, careful physical examination (skin, eye, and muscles) and full body scan (integrated CT-PET) are required in different stages of esophageal cancer. Being responsible for approximately most of EC-related deaths, understanding of EC metastasis dissemination patterns needs more extensive research studies, and these critical data is the cornerstone of optimal cancer approach and treatment.

## Figures and Tables

**Table 1:** Clinicopathological characteristics of EC with unexpected metastases.

Demographics & clinical features	EC with unexpected metastasis
Age at diagnosis (median)	60.7
Gender/male	84%
Gender/female	16%
Histology/SCC	60.5%
Histology/adenocarcinoma	39.5%
Location/upper	8%
Location/middle	27%
Location/lower	65%
Stage I	8
Stage II	9
Stage III	31
Stage IV	52
